# Diseases of the Nose and Throat

**Published:** 1900-09

**Authors:** 


Diseases of the Nose and Throat. By J. Price Brown, M. B., L. R. C. P. E., Member of the College of Physicians and Surgeons of Ontario; Laryngologist to the Toronto Western Hospital; Laryngologist to the Protestant Orphans' Home; Fellow of the American Laryngological, Rhinological and Otological Society; Member of the British Medical Association, the Pan-American Medical Congress, the Canadian Medical Association, the Ontario Medical Association, etc., etc. Illustrated with 159 engravings, including 6 full-page color plates and 9 color-cuts in the text. Pages xvi.—470. Philadelphia: The F. A. Davis Co., Publishers. 1900. [Price, cloth, $3.50.]

This is the first book upon this subject written in Canada, although there are several distinguished teachers and practitioners of laryngology in the Dominion.

The author in his preface tells us that his work is not for the specialist, but for the general practitioner, and while reading the book one cannot fail to appreciate the skill with which he accomplishes his task.

The metric system is adopted throughout the work, an example which might be more widely followed with great propriety. The whole book bears an individual stamp, although the author has followed the classification of diseases given in Bosworth's book on diseases of the nose and throat.

The articles on some diseases are almost exhaustive, too much so perhaps for a work intended for the general practitioner. Among such may be mentioned those on nasal polypi and diseases of the accessory sinuses of the nose. Three exhaustive articles have been written upon subjects which have received special study during the last ten or fifteen years. On the other hand, diseases which were more exhaustively discussed by the older laryngologists, scarcely receive the attention which they deserve.

In some places the author gives exhaustive accounts of recent theories and fails to mention well-known facts of great clinical importance; for example, in the article on paralysis of the larynx he devotes nearly two pages to an account of recent researches in regard to the functions of the adductors and abductors of the cords, and although he mentions that aneurism of the arch of the aorta is a cause of paralysis of the recurrent laryngeal nerve, he fails to tell his readers that the paralysis is, in this case, on the left side of the larynx, because the left recurrent nerve passes around the arch of the aorta and returns from this position to the pharynx—an anatomical fact which they may have forgotten, since they were not reminded of it in the author's description of the anatomy of the larynx. This is a fact that is often of great assistance to the clinician who habitually uses the laryngoscope.

The author fails to lay stress upon the time of life at which congenital syphilis generally occurs, although he incidentally—under the diagnosis of congenital syphilis of the nose writes: "The diagnosis from ordinary purulent rhinitis of childhood should not be difficult, as syphilitic rhinitis will be manifest during early infancy." He fails likewise to mention anywhere in the work syphilis heredetaria tardu, which frequently manifests itself as nasal and pharyngeal lesions. The application of cold externally and the internal or hypodermic administration of pilocarpine are not recommended in the treatment of acute edematous laryngitis.

No mention is made of Asch's and other similar operations on the septum in his description of the treatment of deformities of the nasal septum. It is true such operations can only be performed by the skilled operator, yet all who read systematic works on diseases of the nose should have presented to them a description of these operations with an opinion of the author as to their utility.

The reviewer does not advise the use of menthol, as has the author in the treatment of hay-fever, for it has in his hands proven very irritating to the nasal mucous membrane. One can scarcely understand why the author has omitted to describe in a book on diseases of the nose and throat such a disease as diphtheria, and the same might be said of diseases of the frontal sinus, for in this part of the country, at least, patients with this condition, unless the trouble is so far advanced as to produce exophthalmia, are more apt to consult the rhinologist than the oculist.

The work will compare favorably with the books written upon this subject during the last two or three years in this country and will prove a very useful treatise, we have no doubt, to the class of practitioners in Canada, for which it has been specially written. We have pointed out what seems to us defects, but notwithstanding all these, it is quite as free from sins of omission and commission as the average work of similar character, and is destined to occupy a distinctive place among the recent contributions to the literature of diseases of the upper-air tract. W. S. R.

